# Patient education on PROM completion in clinical care settings: a scoping review

**DOI:** 10.1186/s41687-026-01015-2

**Published:** 2026-02-24

**Authors:** A. Groenewegen, O. M. Biller, J. Greenhalgh, A. Joseph, C. Lindström Egholm, J. Daams, M. Santana, L. Haverman, M. M. van Muilekom, Elizabeth Unni

**Affiliations:** 1 Department of Child and Adolescent Psychiatry and Psychosocial Care, Amsterdam UMC, University of Amsterdam, Emma Children’s Hospital, Amsterdam, The Netherlands; 2 Amsterdam Public Health, Mental Health and Personalized Medicine, Amsterdam, The Netherlands; 3 Amsterdam Reproduction and Development, Child Development, Amsterdam, The Netherlands; 4 Johnson & Johnson, Raritan, NJ USA; 5 School of Sociology and Social Policy, University of Leeds, Leeds, UK; 6 YHEC, University of York, York, UK; 7 REHPA, Danish Knowledge Centre for Rehabilitation and Palliative Care, Odense University Hospital, Nybog, Denmark; 8 Department of Clinical Research, University of Southern Denmark, Odense, Denmark; 9 Amsterdam UMC Location University of Amsterdam, Medical Library, Research Support, Amsterdam, The Netherlands; 10 Department of Community Health Sciences, University of Calgary, Calgary, Alberta Canada; 11 Department of Pediatrics, University of Calgary, Calgary, Alberta Canada; 12 Amsterdam Public Health, Mental Health and Digital Health, Amsterdam, The Netherlands; 13 Department of Social, Behavioral, and Administrative Sciences, Touro College of Pharmacy, New York, NY USA

**Keywords:** Patient-reported outcome measures, Clinical practice, Patient education, Response rates, Implementation

## Abstract

**Background:**

Patient-reported outcome measures (PROMs) are increasingly used in clinical care, and evidence demonstrates they enhance patient care processes and outcomes. However, low PROM completion rates can hinder their use for patient-level monitoring and clinical decision-making. Patient education may help overcome barriers to PROM completion, yet little is known about what education, if any, is offered. The aim of this scoping review was to understand if and how education on PROMs is provided to patients in clinical care.

**Methods:**

This review followed the Preferred Reporting Items for Systematic Reviews and Meta-Analyses extension for Scoping Reviews. MEDLINE, Embase, and APA PsychINFO were searched. Eligible studies used PROMs for individual patient care where PROM results are discussed with the patient, and described patient education on PROMs. Articles were double-screened, data was extracted and relevant data items were categorized and reported.

**Results:**

In total 4392 titles and abstracts were screened, and 292 full texts were reviewed. Only 75 studies were included because they mentioned or described patient education on PROMs. Education was often provided by clinical staff or research staff, using verbal, written or multimodal methods. Education typically occurred prior to PROM completion, and sometimes during PROM completion. The review identified five key components describing the content of education: (1) introduction to PROMs, (2) explanation of their purpose, (3) method of completing PROMs, (4) presentation and (5) utilization of PROM results. These components are presented as the PROMs Patient Education Model. Most studies described to educate on the method of PROM completion, followed by the purpose of PROMs in clinical care. Only a few studies examined patient experiences with education or assessed the impact of education on PROM completion. Despite positive indications, due to the limited number and nature of the included studies, no conclusions on the effect of patient education on PROM completion rates can be made.

**Conclusions:**

While patients may be educated about PROMs in clinical care, it is premature to consider patient education as standard in PROM implementation. This might partly be explained by the lack of detailed reporting in the literature. A survey with healthcare stakeholders could clarify current practices. The effect of patient education on PROM completion remains unclear, and it is recommended that future PROM studies measure the impact of education and report it in literature.

**Supplementary information:**

The online version contains supplementary material available at 10.1186/s41687-026-01015-2.

## Abstract (plain language summary)

Patient-reported outcome measures (PROMs) are standardized questionnaires, used to assess aspects of patient’s health status (e.g. anxiety and pain). PROMs are increasingly being used in clinical care and evidence demonstrates they enhance patient care processes and outcomes. However, low completion rates limit the effectiveness of PROMs. Patient education may be a method to improve completion rates. However, yet little is known about what education, if any, is offered. In this study, we performed a scoping review to understand if and how education is provided to patients on PROMs in clinical care. In total 4392 titles and abstracts were screened, and 292 full texts were reviewed. Of these, only 75 studies were found to mention or describe patient education on PROMs. Education was mainly provided by clinical or research staff using verbal, written, or multimodal methods. Five key components describing the content of education were identified: (1) introduction to PROMs, (2) explanation of their purpose, (3) method of completing PROMs, (4) presentation and (5) utilization of PROM results. The method of completing PROMs (4) was most frequently described by studies. This review indicates that while patients may be educated about PROMs in clinical care, it is premature to consider patient education as standard in PROM implementation. A survey with healthcare stakeholders could clarify current practices. Currently, no conclusions can be made on the effect of patient education on PROM completion, and it is recommended that future PROM studies measure the impact of education and report it in literature.

**Supplementary information:**

The online version contains supplementary material available at 10.1186/s41687-026-01015-2.

## Introduction

Patient-reported outcome measures (PROMs) are increasingly being used in clinical care settings, often completed prior to an appointment through various methods, including self-completion with paper-and-pencil, electronically, and administered by an interviewer. They can be completed solely by the patient, or by a proxy (parent/caregiver) who reports on behalf of the patient, when a patient is incapable of completing the PROMs. Subsequently, PROM results can be communicated to the patient and clinician, using a dashboard when completed electronically, and discussed by the clinician and patient during the clinical appointment. In recent years, PROMs are also increasingly being used for routine monitoring between appointments. In these cases, an automatic alert system is often used based on threshold PROM scores to prompt clinicians (or patients) to discuss treatment, concerns, and/or PROM results in need of attention.

Numerous studies have investigated the effect of using PROMs in daily clinical care, and found improvements in patient-clinician communication, diagnosis and disease notation, disease control, and improvements in quality of life [1, 2]. Despite these benefits, various studies have reported low PROM completion rates, with averages ranging from 25% [3–5] to 55% [6–8]. Not completing PROMs can affect individual patient care by limiting the ability to monitor health outcomes as reported by the patient and incorporate the patient’s perspectives into clinical decision making. Compared to individuals that do complete PROMs, non-completers have been found more likely to come from a minority ethnic or non-White background [9–12], experience greater deprivation [9], social vulnerability [11] and have lower health literacy levels [12, 13], amongst others. Following, a recent systematic review and meta-analysis revealed that patients perceive multiple barriers to PROM completion in clinical care, including a lack of understanding regarding the purpose of the PROMs, a perceived lack of qualification to answer the PROMs, and questions about the relevance of the PROMs in their care [14]. These barriers may influence both the initial willingness to complete PROMs, as well as the actual performance of completing PROMs. One method to address these barriers can be patient education on PROMs.

Patient education is the process of influencing patient behavior and producing changes in knowledge, attitudes, and skills necessary to maintain or improve health [15]. With regards to PROMs, informing patients or proxies about what PROMs are, and why and how they are used in clinical care (changing knowledge and attitudes) may help to overcome the patient’s lack of understanding of the purpose and relevance of PROMs. Instructing patients on how to complete PROMs (change skills) could make patients feel more qualified to complete the PROMs. To support patients after PROM completion, instructions on interpreting PROMs and discussing the PROM results together with the clinician [16], for which the initiative can lie with both the patient and the clinician, can be of value as well. Examples of commonly used procedures and techniques to educate patients include informative pamphlets, videos, (electronically) written and verbal instructions, which can be offered by clinicians, clinic staff and/or researchers.

Based on the literature and from anecdotal evidence, it is often not known whether and how patients (and proxies) are educated about PROMs in clinical care settings [17, 18]. When data is scarce in the literature, a scoping review can be used to explore the extent and nature of literature on a certain topic [19]. Therefore, the goal of this study was to conduct a scoping review to understand if and how education is provided to patients (and proxies) on PROMs in clinical care, including the method, timing, goal and content of education. Second, when available, this study also aimed to describe patients’ and proxies’ opinions on and experiences with education on PROMs and evaluate effects of patient education on PROM completion rates.

## Methods

A scoping review was conducted and reported in accordance with the Preferred Reporting Items for Systematic Reviews and Meta-Analyses extension for Scoping Review (PRISMA-ScR) guidelines [19]. The review protocol was registered on DataverseNL and can be found on https://doi.org/10.34894/BMX15A. The PRISMA-ScR checklist can be found in Additional file 1.

### Eligibility criteria

The eligibility criteria for this review were formulated in terms of context, concept, population [20] and publication characteristics

#### Context

Studies were included when performed in the context of clinical care settings, referring to hospitals, mental health and rehabilitation centers, general practice settings and other outpatient facilities or clinics whose primary purpose is treatment, rehabilitation and care. This includes physical and mental healthcare, both outpatient and inpatient settings. Studies could involve randomized controlled trials (RCTs) and pilot or full-scale implementation studies of PROM use. Studies to develop new PROMs, Core outcome sets (COS) and PROM platforms, as well as validation studies of PROMs and COS without reports on implementation were excluded from the review. Pharmaceutical or medical device research settings were also excluded.

#### Concept

In terms of concepts, the review included studies that either mention or clearly describe patient education on PROMs. In this review, patient education refers to a method to inform patients about what PROMs are and why they are used, give instruction on how to complete PROMs and discuss the PROM results together with the clinician.

In addition, for studies to be included, the use of PROMs in clinical practice must address all of the following aspects:

- The PROMs are used for the individual patient.
- The PROMs are administered (paper-and-pencil, electronically, interview) to and completed by the patient or by a proxy (when a patient is incapable to complete the PROMs due to, for example, age or cognitive difficulties), with the goal to structurally/routinely monitor PROs in a standardized manner.
- The PROM results are made visible and/or communicated to the patient and/or the clinician.
- The clinician discusses the PROM results with the patient, for the purpose of informing the clinical appointment, monitoring, treatment planning, and/or as a source of information for ongoing patient-provider interactions from intake to discharge.

Studies on PROMs for single screening occasions or solely for research purposes (e.g., as a study endpoint) were excluded from the review. Studies in which it was not clear if the PROM met the study’s definition of a PROM were also excluded.

#### Population

Studies meeting the population criteria for review had to involve patients in treatment for any type of health condition(s). Articles about the general population or with healthy samples were excluded from the review. The minimum age criterion for patients to complete PROMs by themselves is (most often) set at eight years [21, 22]. However, we also included articles in which proxies completed the PROMS for patients that cannot complete PROMs themselves (e.g. due to age). Therefore, no limitations were set on the population’s age.

#### Publication specifications

Regarding publication language, only studies published in English were included. No limitations were set on the year of publication. Although PROM use in clinical care became more prominent from 2000 forward, a recent review on the effect of PROM use in clinical care found relevant articles dating back to before 2000 [1]. Finally, reviews, conference proceedings, and full dissertations with multiple articles that are separately published were excluded from the study. No geographic criteria were set.

### Search strategy and information sources

A comprehensive search strategy was developed and performed in February 2024 under guidance of an experienced medical librarian [JD]. The search strategy included three topics: *patient education; PROMs*; and *clinical practice settings* in which PROMs can be used. The first step of the process was the development of a reference set of articles (*N* = 20) that potentially met inclusion criteria for the review. This reference set was subsequently used to identify search terms for the search strategy in an iterative fashion. The reference set consists of articles identified (1) previously by co-authors, (2) via citation tracking of reference lists of relevant articles, (3) via similarity tracking on PubMed, and (4) by using the Connected Papers database to identify other relevant publications (http://www.connectedpapers.com).

The search strategy was initially designed for Ovid Medline, and subsequently translated for additional searches in Ovid Embase, Ovid APA PsycINFO, and Web of Science (WoS) Conference Proceedings Index. The final search strategy is presented in Additional file 2. Note, at a later stage, during pilot screening, a decision was made to exclude conference proceedings from the study due to the concern that conference abstracts miss the level of detail necessary to be eligible for the review.

### Selection of sources of evidence

The identified articles from the searches in Medline, Embase, APA PsycINFO and WoS Conference Proceedings Index were combined and deduplicated. The selection of sources of evidence was conducted using the Rayyan screening software [23].

The title and abstract screening started with a pilot performed by all independent reviewers [AG, OB, JG, AJ, CL, MM, EU], who screened the first 100 titles and abstracts, following the study eligibility criteria. Discrepancies between the reviewers were discussed, and eligibility criteria were adjusted as needed. Thereafter, the reviewers independently screened an additional 50 titles and abstracts, followed by discussion of discrepancies and eligibility criteria. The latter step was repeated until 75% (or greater) agreement was achieved [20]. After reaching sufficient agreement (81% agreement after pilot screening 200 articles), the remaining titles and abstracts were double screened by four reviewer pairs. Based on the limited reports on patient education in the reference set and the first 100 title and abstracts that were screened, it was decided not to apply the eligibility criteria on *patient education on PROMs* during the title and abstract screening, and to only apply this criterion during full text screening. Each full-text article was independently screened by at least two reviewers [AG, OB, AJ, CL, MM, EU]. Reasons for exclusion were documented. Group members who did not perform the screening [MS, LH] were nominated to resolve any disagreements in any of the screening phases.

### Data charting process and data items

The data was extracted from the full text by two reviewers [OB, AJ] and cross-checked by one reviewer [AG], using a predefined data extraction form in Excel. The data extraction form included instructions for data extraction, standard bibliometric information and study specific characteristics (listed below). Before starting the formal data charting process, a pilot test was performed. During the formal data extraction process, group members who did not perform data extraction [MS and LH] were nominated to resolve any disagreements. To document any changes to the data extraction approach, a log was maintained including the change made and by whom.

The following data were collected: study characteristics (including study type, clinical setting, study country, condition of study population, PROM completion rates), context of PROM completion (including mode of PROM completion and mode of discussing PROM results), delivery of patient education on PROMs (including educator, recipient of education, educational method and timing), the goal and content of patient education on PROMs, patients’ and proxies’ opinions on and experiences with education on PROMs, and effect of patient education on PROM completion rates. An extensive item list can be found in Additional file 3.

### Synthesis of results

The results are presented according to the following: (1) study characteristics are described qualitatively, (2) context of PROM completion and (3) delivery of patient education on PROMs are described in percentages as nominal data. The (4) content of patient education is categorized based on components found in literature and subsequently described in percentages as nominal data, (5) opinions and experiences of patient education and (6) the effect of patient education on PROM completion rates are described in text.

### Large language models

To improve spelling and grammar in the scoping review, The large Language Model ChatGPT was used. This tool was exclusively used for language editing, and did not influence the scientific content of this review.

## Results

**Fig. 1:**
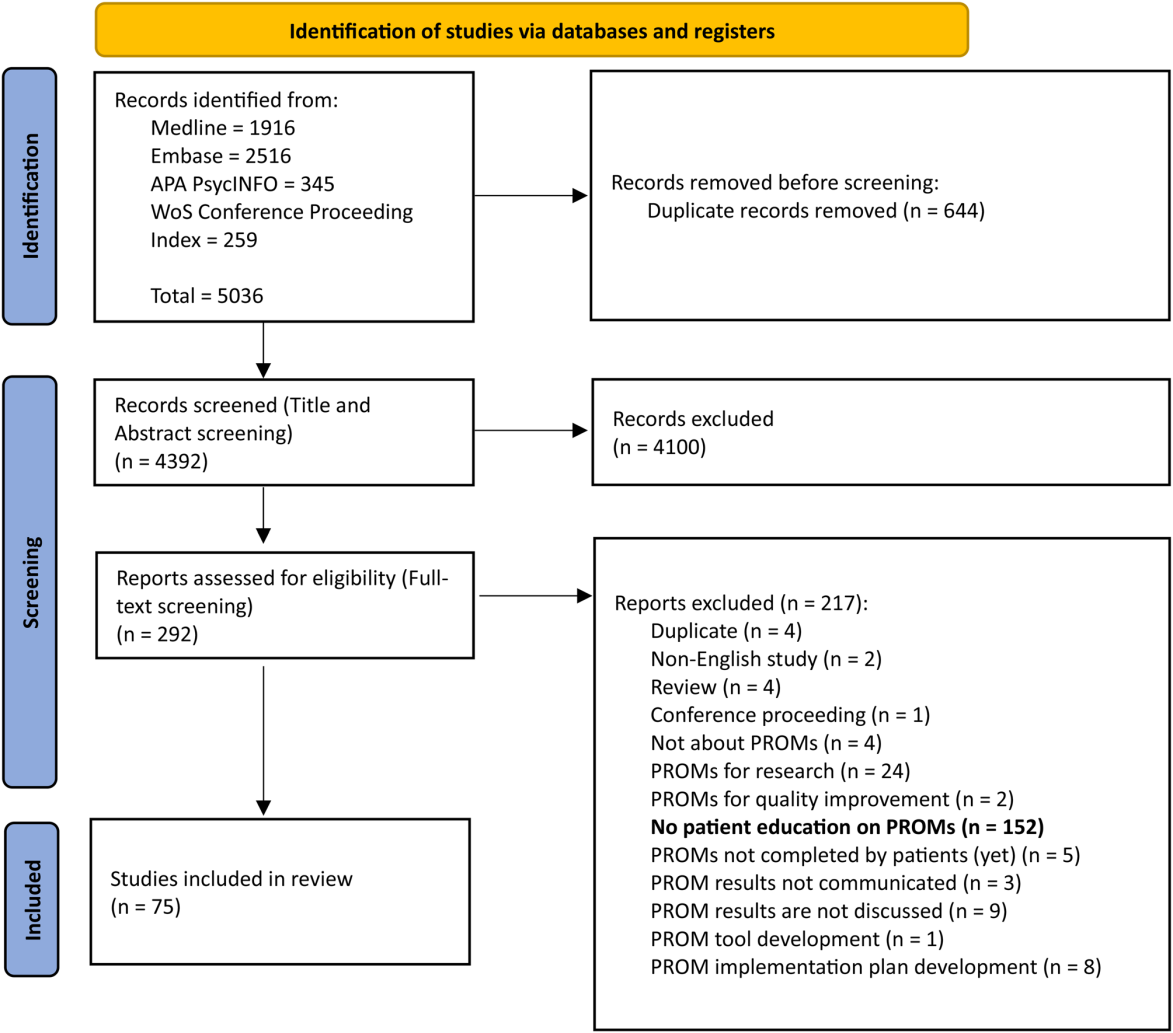
PRISMA flow diagram [24]

Of the 5036 identified records, 75 studies were included in the final review (Fig. 1). The most common reason for excluding identified studies (52%) that did indicate the use of PROMs in daily clinical care in their abstract, was because they did not report on patient education on PROMs in the full-text. Only one included study [25] reported on patient education in the abstract.

### Study characteristics

Table 1 in Additional file 4 presents the characteristics of the included studies. The review includes 75 studies published between 2007 and 2024, that were carried out across seventeen countries, including the United States (*n* = 30), The Netherlands (*n* = 11) and Canada (*n* = 8). Most studies were observational (*n* = 24), implementation (*n* = 22, either describing [*n* = 13] or studying [*n* = 9] PROM implementation), or mixed-method (*n* = 15) studies. Most studies were performed in academic hospitals (*n* = 38) and cancer centers (*n* = 18). The majority of studies included patients with cancer (*n* = 39), and some studies included patients with different conditions (*n* = 9). Commonly, studies included the adult patient population, and only a few the pediatric population (*n* = 11). Most common reported PROMs included Patient-Reported Outcome Measurement Information System (PROMIS) measures (15.6%); European Organization for Research and Treatment of Cancer QLG Core Questionnaire (EORTC-QLQ) (and modules, 14.3%) and the Edmont Symptom Assessment Scale (ESAS, 10.4%) and EuroQol Five Dimension questionnaire (EQ-5D, 10.4%). In most studies, PROMs were used as a source of information for ongoing patient-provider interactions (*n* = 59), and/or for remote monitoring of symptoms (*n* = 31). Finally, 52 studies reported on PROM completion rates. Completion rates were calculated using different methods across studies and ranged between 27% − 100%. Of these 52 studies, 46 studies reported completion rates of 60% or higher. Fifteen articles investigated differences in social determinants of health between completers and non-completers (Additional file 4, Table 2). Most outstanding differences included that PROM completers were more likely to be privately insured (in the United States), have prior computer/internet experience, or be non-Hispanic white.

**Table 1:** Clinical context in which patient education on PROMs is given

	Proportion of studies (%)	Reference numbers of studies
Location of PROM completion		
Remote (e.g. at home)	72%	[26–79]
At the clinic	48%	[65–100]
Mode of PROM completion		
Electronic device (e.g. tablet, mobile phone, computer)	97%	
* External platform*	62%	[26–32, 34, 36–38, 40, 41, 43, 45–48, 50–53, 56–66, 75, 77, 81, 85, 93, 95–98]
* EHR integrated platform*	37%	[33, 39, 42, 44, 49, 54, 67–70, 72–74, 76, 79, 80, 82, 84, 86–90, 92, 94, 99]
* Unknown system*	1%	[100]
Paper & pencil	8%	[46, 55, 71, 83, 91, 93]
Email	4%	[71, 83]
Interview	7%	[42, 45, 50, 51, 65]
Mode of discussing PROM results		
Appointment at clinic	71%	[28, 35, 37, 38, 40–42, 44, 46, 49, 53, 56, 59–78, 80–100]
Telephone appointment	4%	[55, 65, 75]
PROM discussed as needed triggered by threshold score, for remote monitoring	29%	[26, 27, 29, 32–35, 38–40, 43, 47, 48, 50–54, 57, 63, 65, 66, 76]
Not initiated by clinician	4%	[30, 31, 36]
Not specified	4%	[45, 58, 79]

Note. Electronic health record, EHR; Patient-reported outcome measures, PROMs. Percentages are based on reports from studies (*N* = 75) included in this review. Percentages do not add up to 100%, as some studies reported several options

**Table 2:** Delivery of patient education on PROMs

	Proportion of studies (%)*	Reference numbers of studies
**Recipient of education**		
Patient	100%	All studies
Caregiver/partner/family	15%	[28, 32, 41, 48, 56, 60–62, 85, 86, 93]
**Educator**		
Clinician (doctor, nurse)	11%	[29, 30, 37, 38, 42, 44, 83, 89]
Clinical staff	37%	[35, 36, 47, 50, 51, 53, 54, 57, 59, 61–65, 67, 70, 72, 73, 77, 79, 80, 82, 84, 86, 92, 94, 96, 100]
Research staff	32%	[28, 31, 32, 40, 43, 45, 46, 48, 52, 56, 60, 66, 71, 74, 78, 81, 85, 88, 90, 91, 95, 97–99]
Other**	3%	[27, 58]
Not specified	17%	[26, 33, 34, 39, 41, 49, 55, 68, 69, 75, 76, 87, 93]
**Educational method**		
Verbal	76%	[26, 27, 29–32, 34–36, 38, 40, 42–48, 50–54, 56, 57, 59, 63–67, 70–75, 77, 79–86, 88–92, 94–98, 100]
Flyer/guideline	43%	[26, 28, 30, 37, 39–41, 43–46, 48, 49, 51, 55, 56, 60–64, 67, 73, 74, 79, 80, 87, 89, 91–94]
Video	17%	[33, 41, 44, 49, 58, 60–62, 69, 75, 87, 94, 99]
Within platform	16%	[31, 33, 35, 36, 44, 48, 50, 58, 63, 68, 76, 94]
Support with completion	4%	[31, 44, 82]
Other	1%	[78]
**Timing of education**		
Prior to PROM completion	57%	[26–34, 39–43, 46, 47, 49, 51–57, 59, 61, 66, 69, 74–76, 78, 81–84, 86, 87, 93, 94, 96, 98, 99]
Prior to and during PROM completion	32%	[35, 36, 44, 45, 48, 50, 58, 60, 63–65, 71–73, 77, 79, 80, 88–90, 92, 95, 97, 100]
Prior to and after PROM completion	4%	[67, 70, 91]
During PROM completion	1%	[68]
After PROM completion	1%	[85]
Not reported	4%	[37, 38, 62]
**Frequency of education**		
Once, prior to first assessment	33%	[27–30, 32, 34, 39, 42, 43, 47, 50–52, 54, 56, 57, 59, 66, 78, 82, 83, 86, 96, 99]
Prior to every assessment	13%	[40, 45, 49, 55, 70, 76, 89, 92–94]
Prior to first assessment and referred to when needed	39%	[26, 31, 35, 36, 41, 44, 46, 48, 53, 61, 63–65, 67, 71–75, 77, 79–81, 88, 90, 95, 97, 98, 100]
Always available, referred to when needed	7%	[37, 58, 60, 62, 68]
Not specified	8%	[33, 38, 69, 84, 85, 87]

Note. Patient-reported outcome measures, PROMs. *Not all percentages add up to 100% as in some studies multiple recipients, educators, or educational method were applicable. **Other includes volunteers (Barbera & Moody 2019); local personnel (Bash et al. 2020); Nefrovisie (Sipma et al. 2023)

### Context of PROM completion

Table 1 presents the clinical context of studies reporting on patient education on PROMs. Remote completion of PROMs was commonly reported (72%), and 20% of studies offered completing PROMs remotely as well as at the clinic. Almost all studies (97%) offered the PROMs via an electronic device, either via an external platform (62%) or as an application within the electronic health record (EHR) of the health care system (37%). Names of external platforms used include the KLIK PROM platform (*n* = 6), CHES (*n* = 5), Ambuflex (*n* = 2), PatientViewpoint (*n* = 2), and STAR (*n* = 2) amongst others. EHR-integrated platforms include Epic-MyChart (*n* = 7) and Carevive (*n* = 2), amongst others. PROM results were most often discussed during an appointment at the clinic (71%), or as needed following an alert, triggered by a PROM threshold score (29%).

### Delivery of patient education on PROMs

Table 2 presents characteristics of the delivery of patient education on PROMs. In 15% of studies, caregivers/partners/family members received education. Education was most commonly provided by clinical (37%) or research (32%) staff. In 17% of the studies, the educator was not specified. Patient education was commonly offered verbally (76%), and/or by use of guidelines or flyers either provided on paper or online (43%). In three studies (4%), educators helped the patients with the initial PROM completion. Multimodal education (using two or more educational methods) was reported by 47% of studies.

In the majority of studies, education was offered prior to PROM completion (57%) and in multiple studies during PROM completion as well (32%). Four studies (5%) reported offering education after the PROMs were completed. Finally, in most studies, education was given prior to the first assessment time point (72%) and in 46% of studies, individuals could refer to the educational material when needed.

### Goal and content of patient education on PROMs

**Fig. 2:**
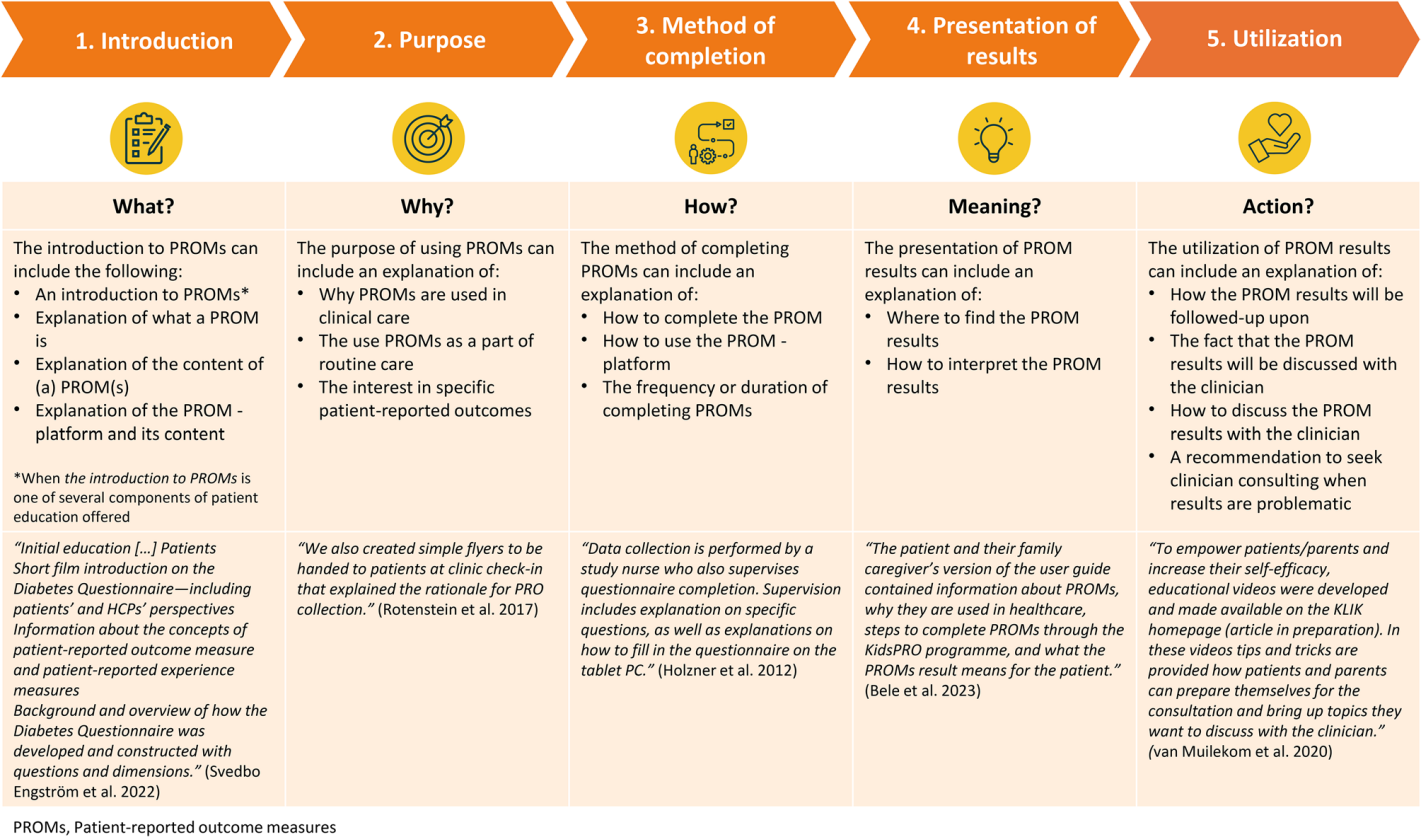
PROMs, Patient-reported outcome measures patient education Model - The content of patient education described by five key components identified in the literature (*N* = 75), including example reports. Examples are taken from studies reporting on three or more components of patient education, with an explicit description of at least one patient education component

The goal of patient education on PROMs was not reported in detail or in an explicit manner in most studies. When it was stated, the goal was centered around the content of education given. When analyzing the included articles on the content of education, we identified five key components: [1] introduction to PROMs, [2] explanation of the purpose of PROMs, [3] method of completing PROMs, [4] presentation of PROM results, and [5] utilization of PROM results. The conceptualization of the key components is based on the findings from the studies included in this review as depicted in Fig. 2.

Table 3 presents the prevalence of the key components of patient education on PROMs amongst the included studies (*N* = 75). Most studies reported to educate patients about the method of PROM completion (65%), followed by the purpose of using PROMs (39%). Only 9% of studies reported on the presentation of PROM results. Twelve studies reported three components of patient education, two studies [44, 87] reported four components and one study [60] reported on all five components of patient education. Examples of reports on patient education can be found in Fig. 2.

**Table 3:** Prevalence of five key components of patient education on PROMs amongst the included studies (*N* = 75)

Component	Proportion of studies (%)	Reference numbers of studies
Introduction to PROMs	21%	[30, 35–37, 42–45, 58, 60, 68, 69, 75, 79, 94, 97]
Purpose of PROMs	39%	[28, 37, 38, 41, 42, 44, 45, 48, 49, 53–55, 58, 60, 61, 64, 67, 68, 72, 73, 79, 83, 86–88, 93, 94, 97, 100]
Method of completing PROMs	64%	[26–32, 34, 35, 37, 40–50, 52–54, 57, 59–61, 63, 66, 68–70, 72–75, 77, 81, 82, 86, 87, 89, 90, 94, 96, 98, 100]
Presentation of PROM results	11%	[28, 52, 60, 74, 75, 85, 87, 91]
Utilization of PROM results	25%	[26, 32, 34, 40, 43, 44, 55, 58, 60–63, 66, 73, 85–87, 91, 93]
No components described	17%	[33, 39, 51, 56, 65, 76, 78, 80, 84, 92, 95, 98, 99]

Note. Patient-reported outcome measures, PROMs

In thirteen articles (17%) none of the described components of patient education were reported. Descriptions within these thirteen articles did indicate that patient education on PROMs was offered, but no additional details were provided. These descriptions include: “*In the chat, patients see a brief introductory video and […]*” [33]; “*The front desk staff […] provided patients with the tablet along with instructions to fill out the RAPID3 questionnaire while in the waiting room*.” [84]; “*Information brochures were provided, and the symptom monitoring program was presented to the patients*.” [51]

Nineteen articles (25%) reported that patients could request assistance when needed. Out of these nineteen articles, one article did not describe any component of patient education but described offering ‘an information sheet’ [92]. Assistance was described with more (‘technical assistance’, assistance with the tablet’, ‘help navigating through the system’) or less (just ‘assistance was provided’, ‘assistance with completion’) detail.

### Opinions and experiences of patient education on PROMs

In four studies [28, 31, 83, 95], patients shared their opinions about and experiences with the education they received on PROMs. The content of patient education differed greatly between studies; however in all studies, patients reported that the education received was helpful or well explained. Moreover, in the study by Biran et al. (2020), in which only the method of PROM completion was explained, patients reported that they did not wish to change the timing or duration of education and felt equipped to use the PROM system following training. However, in the same study, some patients reported feeling overwhelmed by the amount of information offered during the training and patients did not recall the handout they received or reach out to research assistants with questions. Moreover, when reporting on comprehension of the app used, all patients understood how and when to complete the PROMs, however, several patients did not understand that the PROM results would be used in their care, or whether ad hoc symptoms could be reported as well. In the study by Bele et al. (2023) and Depla et al. (2020) patient education focused specifically on the purpose of using PROMs. The majority of patients subsequently reported that they understood the purpose.

In one study (Nordhausen et al. 2022), research staff reported opinions and experiences with education on behalf of the patients, which included appreciation for the personal contact and communication with the research staff who explained the method and supported PROM completion.

In the remaining 70 studies, no direct opinions or experiences with patient education itself were given. However, 37 studies did report on overall experiences with the use of PROMs. These overall experiences could give some indirect indication of the quality of patient education offered. In fifteen studies [27, 52, 53, 57, 58, 60, 63, 66, 69, 74, 75, 77, 84, 95, 98] patients reported that the PROM system was easy to use, and twelve of them reported [27, 52, 53, 57, 60, 63, 66, 69, 74, 75, 77, 98] education on the method of PROM completion. In eight studies [34, 65, 71, 75, 81, 90, 98, 100], patients needed initial assistance in completing PROMs, While seven studies [34, 71, 75, 81, 90, 98, 100] provided education on the method of PROM completion. Six studies [31, 32, 37, 60, 83, 88] reported that not all patients (correctly) understood the goal of using PROMs, though four studies [37, 60, 83, 88] included education on the purpose of using PROMs. In three studies [35, 74, 78], patients reported difficulties with interpreting the PROM results. In none of these studies, the presentation of PROM results was explained. In two other studies [35, 63], patients explicitly reported that it was not clear to them whether their PROM results would be reviewed.

Finally, a few studies made changes in patient education based on the feedback they received in the study. For instance, Snyder et al. (2023) created an interpretation guide, as they experienced that patients had difficulty understanding PROM results. Calligan et al. (2023) added training for patients on how to use the PROM platform on their own device and communicated that clinicians would receive alerts when patients had submitted alarming PROM results. Finally, Chugh et al. (2023) developed an additional educational video specifically for elderly patients, recorded by clinicians, to increase patients’ incentive to complete the PROMs.

### Effect of patient education on PROM completion rates

Only five out of 75 articles reported on the effect of patient education on PROM completion rates. In the clinical trial by Calligan et al. (2023, *N* = 30), an increase from 11/20 (cohort 1) to 9/10 (cohort 2) of patients reaching the 60% completion threshold was found, following four interventions. Two out of four interventions included patient education: training patients on using the PROM platform on their own device (in addition to explaining the PROM system and PROM completion) and informing that the clinician will receive alerts when PROM results reached threshold scores. Following, in the prospective cross-sectional cohort study by Kazazian et al. (2022, *N* = 174) patients that received remote support for the practical aspects of completing and returning virtual PROMs, had higher completion rates than patients that did not receive this support for virtual PROMs (71% versus 44%). In the qualitative study by Viecelli et al. (2022, *N* = 12) clinical staff reported that more patients completed PROMs when staff supported PROM completion. This support could include explaining the purpose and the value of PROM completion, helping to navigate the PROMs, helping to overcome technical difficulties or offering support with completion. In their implementation study, Wagner et al. (2019, *N* = 636) identified that the PROM completion rate was higher amongst patients who read the message in the EHR (Epic-Mychart), which contained instructions and a direct link to complete the PROMs (50% versus 37% involving all patients). Finally, in the implementation study of Wintner et al. (2020, *N* = not reported) a rise in PROM completion was found after the cover letter to patients was expanded with presenting PROMs as a part of routine care, and the staff training was expanded with the rationale, benefits, and solutions for technical issues related to PROM completion (increase from 22% to 83% and later 98%).

## Discussion

This is the first scoping review to examine the extent and nature of reports on patient education on PROMs in clinical care settings. Of the 4392 titles and abstracts screened, only 75 studies were included because they either mentioned or described patient education on PROMs. Education was often provided by clinical staff or research staff, using verbal, written or multimodal methods. Education typically occurred prior to PROM completion, and during PROM completion as well. The review identified five key components describing the content of patient education on PROMs, including: (1) introduction to PROMs, (2) explanation of the purpose of PROMs, (3) method of completing PROMs, (4) presentation of PROM results, and (5) utilization of PROM results, presented as the PROMs Patient Education Model in this review. Studies most frequently described educating patients on the method of PROM completion, followed by education on the purpose of PROMs in clinical care. Only a few studies investigated experiences with patient education on PROMs, or the effect of education on PROM completion rates.

### Lack of reporting on patient education on PROMs in the literature

Overall, the review found a lack of detailed reporting on patient education on PROMs in daily clinical care in the literature. Moreover, in 17% of studies the content of patient education was not clearly described. Given the abundance of articles published on the use of PROMs in daily clinical care, the review shows that it is premature to consider patient education as standard in PROM implementation. However, anecdotally, clinicians and researchers involved in PROM implementation initiatives, do report on offering patient education. Existing efforts of patient education on PROM may thus be underreported in the literature. Therefore, to gain more insight into educational efforts and the benefits of these efforts, the next steps can be to reach out to authors of studies on PROM implementation in clinical care and members of ISOQOL Clinical Practice Special Interest Group (SIG) community to ask about their educational efforts.

### Patient education on PROMs in the literature

The review found that the most common method for patient education was verbal and that almost half of the studies offered multimodal education. Patients could be overwhelmed by information given during appointments, including education on PROMs as reported by Biran et al. (2020). Using multimodal methods might be a approach to support patients in using PROMs, by offering the possibility to revise the educational material at a later stage and catering to different learning styles or abilities. This is in line with recommendations in the literature to supplement verbal instructions with additional educational methods and use multiple educational methods in general [101]. Noteworthy, Biran et al. (2020) further found that patients may experience difficulty recalling to have received written sources of information (e.g. an handout). Therefore, we recommend to remind patients on available educational materials on PROMs continously during their patient journey.

Most studies reported to educate patient on the method of PROM completion. The relative high percentage of studies focussing on this component might by explained by the rise in electronically administered PROMs, for which individuals need to know how to navigate through a platform to access and complete the PROMs. The findings of the current review, however, indicate that explaining the method of completing PROMs is not always suffcient as multiple studies reported that patients still needed initial assistance in PROM completion. Although the current review does not include “support with PROM completion” as a method of patient education, it may be a succesful strategy to support patients as shown by Suri et al. (2022) and Viecelli et al. (2022). However, not all clinical practices have staff available to support patients with PROM completion, and in many instances, PROMs are completed remotely. Therefore, alternative methods including the use of videos, or remote support or Artificial Intelligence-assisted support should be explored.

In addition to patients feeling uncertain about their ability to complete PROMs, a lack of understanding of the purpose or relevance of PROMs is another barrier for PROM completion reported in the literature [14]. The current review identified few studies reporting to educate patients on the purpose of using PROMs. Moreover, despite these efforts, in some studies, the purpose of using PROMs was still not clear or misinterpreted as being used for clinical research or adminstrative needs [83, 88]. Interestingly, only a quarter of studies reported explaining to patients how their PROM results will be used (e.g. discussed during the clinical appointment), and only one study explained where to find or how to interpret the PROM results. Other qualitative studies have also demonstrated that it is often unclear to patients how the PROMs data will be used [2, 88]. Clear descriptions of PROM results presented to patients is important to support their interpretation and subsequent use in clinical care [102].

### Patient education and PROM completion rates

Limited studies investigated the effect of patient education on PROM completion rates. Moreover, the studies that did report on this matter varied greatly in study design, study population, content and method of education. However, the studies do indicate that in general, patient education may be a helpful method to improve completion rates and that education should focus on aspects beyond simply “instructing” individuals to complete PROMs.

Overall, the 35 studies included in this review which reported completion rates observed relatively high PROM completion, which raises the question whether this can be attributed to patient education on PROMs. Earlier studies and some studies included in this review, have identified that patients of a minority ethnic or non-White background or with lower health literacy are more likely to not complete PROMs. Based on the studies within this review, it is not possible to conclude a different impact of patient education on these patient populations. However, as pointed out in the literature [101] these findings do highlight that educational material should be culturally sensitive, contain an appropriate reading level, and contain visual aids for interpretation.

### Strengths and limitations

This scoping review appears to be the first review examining the extent and nature of reports on patient education on PROMs in clinical care settings. The international and multi-disciplinairy background of the project group and the extensive experience in PROM implementation, contributed to the development of a comprehensive search strategy. However, some limitations do exist. During the development of the search string, known PROM initiatives were included, however, it is possible that some intiatives may have been missed. Following, although we did review external links to educational material, we did not contact authors of studies when links where not provided or patient education was not clearly defined. Additionally, the review only includes studies published in peer-review journals. Educational efforts reported elsewhere are therefore missed.

### Implications

The review brings up the question of the impact of patient education on PROM completion and more importantly, its use during the clinical appointment. It is recommended that future PROM implementation and feasibility studies report on patient education on PROMs and measure the impact of patient education on PROM completion rates and its utility during clinical appointments in improving health outcomes. Additionally, patient education should become a standard item to report in studies concerning PROMs in clinical care [103]. Attention to different educational methods, the inclusion of the five domains as described in the PROMs Patient Education Model, and accounting for differences in social determinants of health of the target population is recommended when preparing educational interventions. Following, education is only one strategy to increase PROM completion rates, and more research is needed on the effect of other strategies such as adminstration modalities and reminders [104]. Finally, by improving PROM completion rates, one not only improves individual patient care, but also reduces biases in aggregated PROM data and thus group comparisons for evaluating treatment interventions, which may affect policy-makers and patients in a positive manner [105].

## Conclusion

This scoping review contributes to knowledge by highlighting the limited reporting of patient education in studies using or implementing PROMs in clinical practice. The effect of patient education on PROM completion remains unclear, and it is recommended that future PROM studies measure the impact of education and report it in literature. Also, attention should be given to differences in social determinants of health whilst developing and studying the impact of patient education on PROM completion. Enhancing PROM completion is important to allow the benefits of using PROMs for individual patient care and healthcare policy improvements.

## Electronic supplementary material

Below is the link to the electronic supplementary material.

Supplementary Material 1

Supplementary Material 2

Supplementary Material 3

Supplementary Material 4

## Data Availability

The datasets used and/or analyzed during the current study are available from the corresponding author on reasonable request.

## Abbreviations

COS: Core outcome set
EHR: Electronic health record
PRISMA-ScR: Preferred Reporting Items for Systematic Reviews and Meta-Analyses extension for Scoping Review
PROM: Patient-reported outcome measures
RCT: Randomized controlled trials
SIG: Special Interest Group
WoS: Web of Science
