# Supplementary material for: Patient education on PROM completion in clinical care settings: a scoping review
Source: J Patient Rep Outcomes. 2026 Feb 24;10:37. doi: 10.1186/s41687-026-01015-2 (PMC12963569; doi:10.1186/s41687-026-01015-2)
Supplement: Supplementary file 2 — Supplementary Material 2 [file 41687_2026_1015_MOESM2_ESM.docx]

**Additional file 2**

Final search strategy

|  | **"Ovid MEDLINE(R) ALL <1946 to February 09, 2024>**  **Search date: 12 February 2024"** |  |
| --- | --- | --- |
| **#** | **Searches** | **Results** |
|  | Patient education |  |
| 1 | (education* or engage* or (patient? adj3 (guideline? or guidance)) or instruct* or support or inform or train* or patient activation or learning?).ab,kf,ti. | 3188031 |
| 2 | (email? or poster? handout? or webpage? or video? or pamphlet? or telephone call? or tablet? or text message? or e-mail? or digital tool? or display or dashboard?).ab,kf,ti. | 446604 |
| 3 | coproduction.ab,kf,ti. | 898 |
| 4 | (cross-sectional interview-based survey? or patient self-management resource? or "user friendly data displays for patients" or self-management advice or ((guide or guidance) adj3 (user? or patient?)) or information material).ab,kf,ti. | 13984 |
| 5 | ((complete or completion) adj10 (patient report* or assess* or questionnaire? or inventor* or survey?)).ab,kf,ti. | 59275 |
| 6 | or/1-5 [education] | 3588192 |
|  | Patient-reported outcome measures |  |
| 7 | patient reported outcome measures/ | 14510 |
| 8 | (epro or pro or eprom? or prom? or patient reported outcome?).ab,kf,ti. | 300625 |
| 9 | 7 or 8 | 303964 |
| 10 | (implement* or usability or develop or test or an electronic symptom management program or compliance or compliant).mp. | 3513913 |
| 11 | 9 and 10 [PRO implementation] | 35945 |
|  | Clinical practice settings |  |
| 12 | (clinical practice or clinical care or cancer practice or oncology practice or healthcare).ab,kf,ti. | 643776 |
| 13 | ((routine or standard*) adj3 (report* or collect* or administ*)).ab,kf,ti. | 41127 |
| 14 | ((monitor* or standard*) adj3 (epro? or pro or patient report* or approach* or symptom?)).ab,kf,ti. | 39191 |
| 15 | ((routine or standard) adj3 care).ab,kf,ti. | 89834 |
| 16 | (consultation room? or clinical consultation? or clinical visit?).ab,kf,ti. | 4123 |
| 17 | or/12-16 [clinical setting] | 795158 |
| 18 | and/6,11,17 | 1730 |
| 19 | ("Improving Patient Experience and Health Outcome Collaborative" or iPEHOC or KLIK or Ambuflex or Kids PRO or eRAPID or PatientViewPoint or TickiT or esas or (STAR and symptom?)).mp. | 3159 |
| 20 | 9 and 19 | 215 |
| 21 | 18 or 20 | 1916 |

|  | **"Ovid Embase Classic+Embase <1947 to 2024 February 09>**  **Search date: 12 February 2024"** |  |
| --- | --- | --- |
| **#** | **Searches** | **Results** |
|  | Patient education |  |
| 1 | (education* or engage* or (patient? adj3 (guideline? or guidance)) or instruct* or support or inform or train* or patient activation or learning?).ab,kw,ti. | 4166409 |
| 2 | (email? or poster? handout? or webpage? or video? or pamphlet? or telephone call? or tablet? or text message? or e-mail? or digital tool? or display or dashboard?).ab,kw,ti. | 627419 |
| 3 | coproduction.ab,kw,ti. | 942 |
| 4 | (cross-sectional interview-based survey? or patient self-management resource? or "user friendly data displays for patients" or self-management advice or ((guide or guidance) adj3 (user? or patient?)) or information material).ab,kw,ti. | 23006 |
| 5 | ((complete or completion) adj10 (patient report* or assess* or questionnaire? or inventor* or survey?)).ab,kw,ti. | 100952 |
| 6 | or/1-5 [education] | 4728455 |
|  | Patient-reported outcome measures |  |
| 7 | *patient reported outcome/ | 17893 |
| 8 | (epro or pro or eprom? or prom? or patient reported outcome?).ab,kw,ti. | 454592 |
| 9 | 7 or 8 | 456672 |
| 10 | (implement* or usability or develop or test or an electronic symptom management program or compliance or compliant).mp. | 6190603 |
| 11 | 9 and 10 [PRO implementation] | 80082 |
|  | Clinical practice settings |  |
| 12 | (clinical practice or clinical care or cancer practice or oncology practice or healthcare).ab,kw,ti. | 952850 |
| 13 | ((routine or standard*) adj3 (report* or collect* or administ*)).ab,kw,ti. | 62971 |
| 14 | ((monitor* or standard*) adj3 (epro? or pro or patient report* or approach* or symptom?)).ab,kw,ti. | 57039 |
| 15 | ((routine or standard) adj3 care).ab,kw,ti. | 159095 |
| 16 | (consultation room? or clinical consultation? or clinical visit?).ab,kw,ti. | 7318 |
| 17 | or/12-16 [clinical setting] | 1199857 |
| 18 | and/6,11,17 | 3625 |
| 19 | ("Improving Patient Experience and Health Outcome Collaborative" or iPEHOC or KLIK or Ambuflex or Kids PRO or eRAPID or PatientViewPoint or esas or TickiT or (STAR and symptom?)).mp. | 6030 |
| 20 | 9 and 19 | 490 |
| 21 | 18 or 20 | 4037 |
| 22 | limit 21 to "remove medline records" | 2516 |

|  | **Ovid APA PsycInfo <1806 to February Week 2 2024> Search date: 12 February 2024** |  |
| --- | --- | --- |
| **#** | **Searches** | **Results** |
|  | Patient education |  |
| 1 | (education* or engage* or (patient? adj3 (guideline? or guidance)) or instruct* or support or inform or train* or patient activation or learning?).ab,id,ti. | 1818681 |
| 2 | (email? or poster? handout? or webpage? or video? or pamphlet? or telephone call? or tablet? or text message? or e-mail? or digital tool? or display or dashboard?).ab,id,ti. | 121771 |
| 3 | coproduction.ab,id,ti. | 350 |
| 4 | (cross-sectional interview-based survey? or patient self-management resource? or "user friendly data displays for patients" or self-management advice or ((guide or guidance) adj3 (user? or patient?)) or information material).ab,id,ti. | 1967 |
| 5 | ((complete or completion) adj10 (patient report* or assess* or questionnaire? or inventor* or survey?)).ab,id,ti. | 19316 |
| 6 | or/1-5 [education] | 1898541 |
|  | Patient-reported outcome measures |  |
| 7 | patient reported outcome measures/ | 858 |
| 8 | (epro or pro or eprom? or prom? or patient reported outcome?).ab,id,ti. | 25906 |
| 9 | 7 or 8 | 25981 |
| 10 | (implement* or usability or develop or test or an electronic symptom management program or compliance or compliant).mp. | 1136523 |
| 11 | 9 and 10 [PRO implementation] | 6792 |
|  | Clinical practice settings |  |
| 12 | (clinical practice or clinical care or cancer practice or oncology practice or healthcare).ab,id,ti. | 129450 |
| 13 | ((routine or standard*) adj3 (report* or collect* or administ*)).ab,id,ti. | 8157 |
| 14 | ((monitor* or standard*) adj3 (epro? or pro or patient report* or approach* or symptom?)).ab,id,ti. | 6097 |
| 15 | ((routine or standard) adj3 care).ab,id,ti. | 10480 |
| 16 | (consultation room? or clinical consultation? or clinical visit?).ab,id,ti. | 914 |
| 17 | or/12-16 [clinical setting] | 151569 |
| 18 | and/6,11,17 | 314 |
| 19 | ("Improving Patient Experience and Health Outcome Collaborative" or iPEHOC or KLIK or Ambuflex or Kids PRO or eRAPID or PatientViewPoint or esas or TickiT or (STAR and symptom?)).mp,tm. | 680 |
| 20 | 9 and 19 | 42 |
| 21 | 18 or 20 | 345 |

|  | **Thomson ISI Web of Science Search date: 12 February 2024** |  |
| --- | --- | --- |
| **#** | **Searches** | **Results** |
|  | Patient education |  |
| 1 | TS=(education* or engage* or (patient? NEAR/2 (guideline? or guidance)) or instruct* or support or inform or train* or "patient activation" or learning?) | 6370872 |
| 2 | TS=(email? or poster? handout? or webpage? or video? or pamphlet? or telephone call? or tablet? or "text message?" or "e-mail?" or "digital tool?" or display or dashboard?) | 1095867 |
| 3 | TS=coproduction | 5194 |
| 4 | TS=("cross-sectional interview-based survey?" or "patient self-management resource?" or "user friendly data displays for patients" or "self-management" advice or ((guide or guidance) NEAR/2 (user? or patient?)) or "information material") | 16595 |
| 5 | TS=((complete or completion) NEAR/9 ("patient report*" or assess* or questionnaire? or inventor* or survey?)) | 162249 |
| 6 | #1 OR #2 OR #3 OR #4 OR #5 | 7413981 |
|  | Patient-reported outcome measures |  |
| 7 | TS=(epro or pro or eprom? or prom? or "patient reported outcome?") | 398654 |
| 8 | TS=(implement* or usability or develop or test or an "electronic symptom management program" or compliance or compliant) | 12283565 |
| 9 | #7 AND #8 | 95479 |
|  | Clinical practice settings |  |
| 10 | TS=("clinical practice" or "clinical care" or "cancer practice" or "oncology practice" or healthcare) | 676782 |
| 11 | TS=((routine or standard*) NEAR/2 (report* or collect* or administ*)) | 47821 |
| 12 | TS=((monitor* or standard*) NEAR/2 (epro? or pro or "patient report*" or approach* or symptom?)) | 57625 |
| 13 | TS=((routine or standard) NEAR/2 care) | 95461 |
| 14 | TS=("consultation room?" or "clinical consultation?" or "clinical visit?") | 2093 |
| 15 | #10 OR #11 OR #12 OR #13 OR #14 | 855761 |
| 16 | #6 AND #9 AND #15 | 2516 |
| 17 | TS=("Improving Patient Experience and Health Outcome Collaborative" or iPEHOC or Ambuflex or "Kids PRO" or eRAPID or PatientViewPoint or TickiT or klik or esas or (STAR and symptom?)) | 4142 |
| 18 | #7 AND #17 | 222 |
| 19 | #6 AND #9 AND #15 and Conference Proceedings Citation Index – Social Science & Humanities (CPCI-SSH) or Conference Proceedings Citation Index – Science (CPCI-S) (Web of Science Index) | 37 |
| 20 | #18 OR #19 | 259 |
