# Supplementary material for: Patient education on PROM completion in clinical care settings: a scoping review
Source: J Patient Rep Outcomes. 2026 Feb 24;10:37. doi: 10.1186/s41687-026-01015-2 (PMC12963569; doi:10.1186/s41687-026-01015-2)
Supplement: Supplementary file 3 — Supplementary Material 3 [file 41687_2026_1015_MOESM3_ESM.docx]

**Additional file 3**

**Data items**

1. Researcher name who completed the data extraction
2. **Study reference**
3. Authors
4. Year of publication
5. Title
6. Journal
7. Volume
8. Issue
9. Article number
10. Pages
11. Digital Object Identifier
12. **Study details**
13. Corresponding author(s) contact details
14. Duration of the study (start/end dates)
15. **Study methods**
16. Study aim(s)/objective(s)
17. Study type (implementation science study, observational study, quality study, mixed-methods study, clinical trials, quality improvement study)
18. Study design (general procedures and methods used in the study) and outcomes
19. Method(s) of recruitment and /or invitation to Patient-reported outcome measure (PROM) completion

**D. Context**

1. Country/countries where the study was conducted
2. Study setting(s) (specific clinical setting when available)
3. Institution name(s)
4. PROM completion rates
5. Method of measuring PROM completion rates

**E.** **Population**

1. Size of target population (when available)
2. Number of patients invited to complete PROM(s)
3. Number of patients that received PROMs
4. Baseline sample characteristics (when available: age (range), sex, race/ethnicity, educational level, other)
5. Disease state of patient population

**F. Concept**

**Context of PROM completion**

1. Goal of using PROMs in clinical practice (e.g. source of information for ongoing patient-provider interactions, remote symptom monitoring)
2. PROM(s) used in the study
3. Location of PROM completion (e.g. remote, at the clinic, both)
4. Mode of PROM administration (e.g. paper, electronic, interview)
5. Name of PROM platform (when applicable)
6. PROM completer(s) (e.g. patient, proxy, both, or varies throughout study)
7. PROM administration schedule (frequency and timing)
8. Mode of discussion on PROM(s) results (e.g. appointment at the clinic, telephone appointment)

**Aim 1: Mode, timing, goal and content of patient education on PROMs**

1. Recipient of education
2. Educational methods used (e.g. verbal instructions, written instructions, video)
3. Educators) (e.g., clinicians, clinic staff, research staff)
4. Frequency of education
5. Duration of education
6. Timing of education on PROM(s) in relation to patient’s clinical care (e.g. during or after the initial appointment)
7. Goal of education
8. Content of education
9. Location of education in the article
10. Other

**Aim 2: Opinions and experiences with PROM education**

1. Recipient’s opinions on PROM education (e.g. preferences, usefulness, acceptability)
2. Recipient’s experiences with PROM education (e.g. understandability, feelings, interpretability)
3. The reporter of the opinion or experience from the recipient of education (e.g. clinician, patient, caregiver).

**Aim 3: Effect of patient education on PROM completion rates**

1. Effect of patient education on PROM completion rates

**Other**

1. Other quantitative outcomes following from patient education on PROMs (e.g. self-efficacy)
2. Future directions regarding patient education on PROMs
3. Key terms (used in abstracts and/or in text that are novel and important for describing patient education on PROMs)

**G. Other**

34. Data that does not fit into the extraction template (requiring discussion)

36. Other questions to the team

37. General notes
