# Supplementary material for: Patient education on PROM completion in clinical care settings: a scoping review
Source: J Patient Rep Outcomes. 2026 Feb 24;10:37. doi: 10.1186/s41687-026-01015-2 (PMC12963569; doi:10.1186/s41687-026-01015-2)
Supplement: Supplementary file 4 — Supplementary Material 4 [file 41687_2026_1015_MOESM4_ESM.docx]

**Additional file 4**

**Table 1:** Study characteristics

| First author, year of publication | Study type | Clinical setting(s) | Study country | Number of individuals receiving PROMs, or otherwise specified | Condition | Age | Purpose of PROM use* | PROM completion rates |
| --- | --- | --- | --- | --- | --- | --- | --- | --- |
| Astradsson et al. 2023 | Implementation description | Academic Hospital | Denmark | 227 | Hydrocephalus | 15+ years | A, B | NR |
| Baeksted et al. 2017 | Mixed Method | Academic hospital | Denmark | 54 | Prostate cancer | 51–88 years, Md=69 years | A | 97% PROMs of expected |
| Bamgboje-Ayodele et al. 2022 | Implementation science study | Hospital | Australia | NR | Lung cancer | NR | A, B | NR |
| Barbera & Moody 2019 | Implementation description | Cancer Center | Canada | 30,000–40,000 unique responses/month | Cancer various | NR | A | NR |
| Basch et al. 2007 | Mixed Method | Cancer Center | United States | 107 | Lung cancer | 18–70+ years, Md=62 years | A, B | 78%​ overall adherence rate |
| Basch et al. 2020 | Clinical trials | Cancer Center | United States | 497 (intervention: 26, control: 26) | Cancer various | 29-89 years, Md=63 years | A, B | 92% of expected weekly reports |
| Bele et al. 2023 | Observational | Academic hospital | Canada | 5 children  4 family/ caregivers | Asthma​ | 8–18 years​ | A | NR |
| Benavent et al. 2022 | Mixed Method | Academic hospital | Spain | 46 | Arthritis | RA: M=48 years, SD=12  SpA: M=42 years, SD=9 | B, D | 80% patients submitted at least one entry |
| Benze et al. 2019 | Clinical trials | Cancer Center | Germany​ | 40 | Advanced solid cancer | 27–73 years, M=57 years, SD=12 | B | 70% for daily assessments 87% for weekly assessments |
| Bhat et al. 2020 | Implementation description | Academic hospital | United States | 200400 | Total joint arthroplasty | 17-95 years, M=66 years | A | 62% preoperative completion 53% postoperative completion |
| Biber et al. 2018 | Implementation science study | Academic hospital | United States | 200000 | Various | NR | A | 47% completed an assessment |
| Biran et al. 2020 | Mixed Method | Cancer Center | United States | 9 | Multiple myeloma | 56–76 years, M=69.7 years, SD=6.5​ | A, B | 83% completion rate for overall weekly guided sessions 67% patients completed all 4 weekly guided sessions |
| Brant et al. 2019 | Mixed Method | Cancer Center | United States | 212 | Gynecology and breast cancer | 28–81 years, M=56.3 years | A | NR |
| Bruckel et al. 2015 | Implementation description | Hospital | United States | 474 | Cardiovascular disease | M=61 years​ | A | NR |
| Calligan et al. 2023 | Observational | Hospital | Canada​ | 30 | Pediatric cancer | 8–18 years | A, B | 55% - 90% reached feasibility threshold of >60% completion |
| Chugh et al. 2023 | Observational | Academic hospital | United States | 2933 | Inflammatory Bowel Disease | M=41 years (IQ =32-55) | A, B | 40% completed at least one ePRO module 22% completed at least three ePRO modules over time |
| Cowan et al. 2016 | Observational | Cancer Center | United States | 96 | Gynecologic cancer​ | 18-74 years, Md=55.5 years | A, B | 74% completed at least 4/7 surveys |
| Davis et al. 2020 | Mixed Method | Primary care clinic | Canada​ | 4 | Mental health disorders | NR | A, B | NR |
| De Wit et al. 2019 | Observational | Hospital | The Netherlands | Survey, 72 HCP | Cancer various | Adults | A | NR |
| Depla et al. 2020 | Observational | Academic Hospital | The Netherlands | 66 | Pregnancy & childbirth​ | 28-34 years | A | 97% mean completion |
| Depla et al. 2023 | Mixed Method | Academic Hospital Hospital | The Netherlands | Survey, 159 HCP, Focus groups, 79 HCP | Perinatal care | NA | A, F | NR |
| Desai et al. 2020 | Implementation science study | Hospital | United States | 1,691 | Rheumatoid arthritis​ | M=61.8 years, SD=14.4 | A | 63% completion sustained through January 2019​ |
| Efficace et al. 2022 | Mixed Method | Academic hospital | Italy​ | 190 | Hematologic malignancies​ | 21–91years, Md=57 years​ | A, B | NR |
| Engelen et al. 2010 | Implementation description | Academic hospital | The Netherlands​ | 169 (intervention: 85, control:84) | Pediatric cancer | 0–18 years | A | 100% completion |
| Fridriksdottir et al. 2023 | Clinical trials | Academic hospital | Iceland | 103 | Gastrointestinal and hematologic cancers | 21–89years, M=61 years | B, D | 70% - 99.6%​ |
| Generalova et al. 2021 | Clinical trials | Cancer Center | United States | 144 (intervention: 72, control: 72) | Thoracic, genitourinary, gastrointestinal cancer | 18 years or older | A, B | 72% ePROs were completed |
| Gerhardt et al. 2018 | Observational | Academic Hospital | United States | NR | Various | Children | A | 75% of PROMs |
| Haverman et al. 2014 | Implementation description | Academic hospital | The Netherlands | 1,450 | Various | 0-18years | A | 71% of PROMs |
| Holzner et al. 2012 | Implementation description | Academic Hospital | Austria, Germany, Switzerland, United Kingdom, Denmark, Australia | 5,000 | Various | NR | A | NA |
| Howell et al. 2020 | Mixed Method | Academic Hospital Cancer Center | Canada | 6,000 | Cancer | NR | A, B | 37% - 86% baseline completion |
| Katzan et al. 2011 | Implementation description | Academic Hospital | United States | 123,455 | Various neurological conditions | NR | A | 75% |
| Kazazian et al. 2022 | Observational | Academic Hospital | Canada | 174 | Cancer | 19-90 years, Md=62 years | A, C, G | 44% - 74% of PROMs |
| Kennedy et al. 2022 | Clinical trials | Cancer Center | United Kingdom | 69 (feasibility group: 24, audit group: 45) | Ovarian cancer | Feasibility group: 21.0-78.7 years, Md=62.8 years Audit group: 41.9-85.4 years, Md=62.3 years | B | 75-82% throughout the study |
| Kristensen et al. 2022 | Observational | Mental Health Center | Denmark | Comprehensibility test, N=19 | Schizophrenia and unipolar depression | 18 or older | A | NR |
| Kumar et al. 2017 | Implementation science study | Hospital | United States | 99 | Postoperative ventral hernia repair | NR | B | 36% completion following earlier telephone call 55% completion following later telephone call |
| Lombi et al. 2023 | Observational | Hospital | Italy | Interviews, N=19 patients | Cancer | 35-78 years, M=57 years | A | NR |
| Maas et al. 2024 | Mixed Method | Academic Hospital | The Netherlands | 245 Evaluation survey, N=43 patients Interviews, N=5 patients, N=5 HCP | Cancer in survivorship phase | 18-30years, M=24.35 years, SD=2.98 | A | 90% patients completed PROMs |
| Macaron et al. 2020 | Observational | Academic hospital | United States | 4,199 | Multiple Sclerosis | 40.2–58.8 years, Md=49.9 years | A | 41 -98% completion of PROMs |
| McCleary et al. 2022 | Implementation description | Cancer Center | United States | NR | Cancer | Adults | B, C | 27% completion across institute |
| Medina et al. 2022 | Clinical trials | Cancer Center | Spain | 328 (intervention: 141, control: 48) | Breast cancer | Intervention group: M=52.35 years, S =8.57  Control group: M=55.15 years, SD=9.55 | B, C | 75% use rate of platform |
| Meryk et al. 2021 | Clinical trials | Academic hospital | Austria | 12 | Pediatric cancer | Md=7.2 years (IQ1=6.7; IQ3=10.1) | A, B | 61% Md completion |
| Newman et al. 2012 | Implementation science study | Hospital | United States | NR | Rheumatic diseases | Adults | A | 80% mean completion |
| Nordhausen et al. 2022 | Implementation science study | Cancer Center | Germany | 1597 | Cancer | Reported separately for each PROM in supplemental materials. | A | 85% out of contacts at least 1 PROM completion |
| Palos & Suarez-Almazor 2021 | Implementation description | Cancer Center | United States | NR | Cancer in survivorship | Adults | A | 33% -63% PROM completion rates across institutes |
| Patt et al. 2021 | Implementation description | Cancer Center | United States | 4375 | Cancer | 78% >54years | A, B | 72% to 52% completion rate of PROM sent |
| Porcher et al. 2022 | Observational | Hospital | France | 524 | Receiving oral anticancer agent | 26.0–92.0 years, Md=68.0 years | B | NR |
| Porter et al. 2021 | Clinical trials | Primary care clinic | United Kingdom | 68 | Various | 48-90 years, M=70 years | A | 100% |
| Prasse et al. 2023 | Observational | Academic Hospital | United States | 71 | Postoperative full endoscopic surgery | 22-87years, M=58 years, SD=2 | B | 85% completed at least 1 postoperative PROM 96% completion after implementing reminders in app |
| Rasschaert et al. 2021 | Observational | Academic hospital | Belgium | 168 | Receiving antineoplastic agent(s) | 23-88 years, M=60.69 years | B | NR |
| Rayner et al. 2014 | Observational | Academic hospital | United Kingdom | 926 | Various | Adults | C | 40%-98% completion |
| Rhodes et al. 2024 |  | Academic hospital | United States | 638 children 1461 parents | Pediatric obesity | 50% 12-18 years | A | 94% of parents provided a complete survey 83% of children provided a complete survey |
| Rocque et al. 2022 | Implementation science study | Academic hospital | United States | Pilot PROM completion, N = 70 patients Interviews, N = 12 HCP | Cancer various | 80% = 60-74 years 20% < 74 years | B | 70% completed all PROMs in phase 1 90% of opened administrations were completed |
| Rodrigue et al. 2015 | Observational | Academic hospital | United States | 208 | Post-living kidney donation surgery | M=44.1 years, SD=11.2 | B | 47% - 59% completion |
| Rotenstein et al. 2017 | Mixed Method | Cancer Center | United States | 7640 Surveys, N= 53 HCP Interviews, N= 8 HCP | Cancer patients receiving radiation | Adults | A | NR |
| Schepers et al. 2017 | Implementation science study | Academic hospital | The Netherlands​ | 205 Survey, N = 28 HCP | Pediatric cancer | 0–7 years, M=3.76 years, SD=2.10 8–18 years, M=12.83 years, SD = 2.68 | B, D | 66–85% |
| Shinoda et al. 2022 | Observational | Hospital | Japan​ | 84 | COPDD and Asthma-COPD Overlap | M=68.7 years, SD=9.17​ | B | NR |
| Short et al. 2022 | Mixed Method | Hospital | Canada, United States | 1632 Training evaluation, N = 18 staff and providers Surveys, N = 1,102, 200 patients, N=16 staff and providers | HIV | 50% ≥ 50 years | A | >99% |
| Sipma et al. 2023 | Observational | Other | The Netherlands | Interviews, N= 13 HCP, N=14 patients | End-stage kidney disease | 42 – 85 years | A | NR |
| Snyder et al. 2013 | Mixed Method | Academic Hospital | United States | 52 patients, 11 HCP | Cancer various | 28–81 years, Md=58 years | A | 71% Md completion per patient |
| Sprave et al. 2023 | Clinical trials | Academic Hospital | Germany | 100 (intervention = 50, control = 50) | Head and neck cancer undergoing (chemo)radiotherapy | Intervention: M=60 years Control: M=66 years | A, E | 100% of ePRO group patients answered ≥80% of daily PROM questions |
| Suri et al. 2022 | Observational | Hospital | Canada | 692 | HIV | M=45.6 years, SD=12.3 | A | 97% |
| Svedbo Engström et al. 2022 | Qualitative | Hospital Primary care clinic | Sweden | Focus groups, N = 15 patients, N = 20 HCP | Diabetes (Type 1 & Type 2) | NR | A, H | 39% |
| Sztankay et al. 2019 | Mixed Method | Academic hospital | Austria | 140 Survey, N = 40 patients, N = 15 HCP | Multiple Myeloma | 38–93 years, M=65.4 years, SD=11.8 | A, H | 94% patients provided 748 PRO assessment time points in year 1 |
| Theiss et al. 2023 | Observational | Academic hospital | United States | 549 | Colorectal surgery patients | Md=57 years (Q1–Q3: 43–67) | A | 81% of patients completed at least one PRO survey |
| Trautmann et al. 2016 | Quality improvement | Academic hospital | Germany | 160 | Cancer various | 38–93 years, M=63 years | A | 67% of participants provided complete data on all PRO scales |
| Van Muilekom et al. 2022 | Mixed Method | Academic hospital | The Netherlands | Focus groups, N = 8 patients N= 17 parents Survey, N = 31 patients, N = 130 parents | Various | Focus groups, patients: 13.10-18.8 years, Md=15.3 years; children of parents: 2.1-16.9 years, M=10.4 years Questionnaire, patients: 12.4-19.2 years, Md=15.7 years; children of parents: 0.9-19.1 years, Md=9.3 | A | NA |
| Van Oers et al. 2021 | Implementation science study | Academic Hospital | The Netherlands | N = 8 PROM implementation experts | Various | Pediatric and adults | A | NR |
| Veltkamp et al. 2022 | Observational | Academic hospital | The Netherlands | 121 | Chronic Kidney Disease | M=9.7years, SD=4.8 | A | 79% |
| Viecelli et al. 2022 | Qualitative | Other | Australia​ | Focus groups and interviews, N = 12 patients, N = 29 HCP | Undergoing maintenance hemodialysis | 39-88years, M=69.5years, SD=13.4 | A | NR |
| Wagner et al. 2015 | Implementation description | Cancer Center | United States | 636 | Gynecologic cancers | 21–90 years, M=55.1 years, SD= 12.8 | A | 35% completion among all eligible |
| Warrington et al. 2019 | Observational | Academic hospital | United Kingdom | 12 | Breast cancer undergoing chemotherapy​ | 33-73years, M=47.5 years, SD=10.3 | A, B | 63% average for weekly completion |
| Wintner et al. 2015 | Observational | Academic hospital | Austria | 279 (ePRO: 113, home-ePRO: 45) | Various | Clinic-ePRO group: 22–81 years, M=45.1 years, SD=14.4  Home-ePRO group: 29–74 years, M=58.7 years, SD=10.4 | A, B | NR |
| Wintner et al. 2020 | Implementation description | Rehabilitation Center | Austria | NR | Cancer patients undergoing inpatient rehabilitation​ | NR | A | 90% completion at settlement |
| Wu et al. 2016 | Quality improvement | Academic Hospital | United States | Interviews, N=42 patients, N= 12 HCP | Breast and prostate cancer | 32–83 years, Md=65 years | A | NR |
| Zhang et al. 2019 | Qualitative | Cancer Center | United States | Interviews, N = 11 HCP | Orthopedic and cancer patients | NR | B, C | NR |

Chronic Obstructive Pulmonary Disease, COPD; Health Care Provider, HCP; Human Immunodeficiency Virus , HIV; Inter Quartile, IG; Not reported, NR; Median, Md; Mean, M; Patient-reported outcome, PRO; Patient-reported outcome measure, PROM; Rheumatoid Arthritis, RA; Spondyloarthritis, SpA

*PROM purposes included (A) Source of information for ongoing patient-provider interaction, (B) Remote symptom monitoring, (C) Screening, (D) Support self-management of symptoms,(E) Enhance treatment satisfaction, (F) Quality improvement, (G) Adhere to mandate, (H) Inform data registries.

**Table 2**. Investigated differences between PROM-completers and non-completers.

| **Patient characteristics** | **Investigated by studies, N** | **Difference found, N (%)** | **Found difference** |
| --- | --- | --- | --- |
| Age | 12 | 3 | PROM completers are older(1) ; PROM completers are younger (2, 3) |
| Disease | 8 | 1 | Large variation in PROM completion found across different health services/conditions (4) |
| Sex | 7 | 1 | PROM completers are more likely female(1) |
| Geographic location | 3 | 1 | PROM completion rates differed per geographic region(5) |
| Educational level | 3 | 0 | N/A |
| Insurance | 3 | 2 | PROM completers were more likely (privately) insured in the US only(6, 7) |
| Computer/internet experience | 3 | 2 | PROM completers have more computer or internet experience(1, 8) |
| Marital status | 2 | 1 | PROM completers are more likely married/partnered(1) |
| Work status | 2 | 0 | N/A |
| Ethnicity/race | 2 | 2 | PROM completers were less likely to be from minority race or Black (6, 7) |
| ADI | 1 | 1 | PROM completers have a higher ADI(1) |
| Primary language | 1 | 1 | PROM completers were more likely to have English as primary language(1) |

Area Deprivation Index, ADI; Patient-reported outcome measure, PROM

**References**

1. Chugh R, Liu AW, Idomsky Y, Bigazzi O, Maiorano A, Medina E, et al. A Digital Health Intervention to Improve the Clinical Care of Inflammatory Bowel Disease Patients. Applied Clinical Informatics. 2023;14(5):855-65.

2. Fridriksdottir N, Ingadottir B, Skuladottir K, Zoega S, Gunnarsdottir S. Supportive Digital Health Service During Cancer Chemotherapy: Single-Arm Before-and-After Feasibility Study. JMIR Formative Research. 2023;7:e50550.

3. Macaron G, Moss BP, Li H, Baldassari LE, Rao SM, Schindler D, et al. Technology-enabled assessments to enhance multiple sclerosis clinical care and research. Neurology Clinical Practice. 2020;10(3):222-31.

4. Rayner L, Matcham F, Hutton J, Stringer C, Dobson J, Steer S, et al. Embedding integrated mental health assessment and management in general hospital settings: feasibility, acceptability and the prevalence of common mental disorder. General Hospital Psychiatry. 2014;36(3):318-24.

5. Patt D, Wilfong L, Hudson KE, Patel A, Books H, Pearson B, et al. Implementation of Electronic Patient-Reported Outcomes for Symptom Monitoring in a Large Multisite Community Oncology Practice: Dancing the Texas Two-Step Through a Pandemic. JCO Clinical Cancer Informatics. 2021;5:615-21.

6. Rodrigue JR, Vishnevsky T, Fleishman A, Brann T, Evenson AR, Pavlakis M, et al. Patient-Reported Outcomes Following Living Kidney Donation: A Single Center Experience. Journal of Clinical Psychology in Medical Settings. 2015;22(2):160-8.

7. Theiss LM, Wood L, Shao C, Marques I, Kim DH, Hollis R, et al. Disparities in Perioperative Use of Patient Engagement Technologies - Not All Use is Equal. Annals of Surgery. 2023;277(1):e218-e25.

8. Basch E, Iasonos A, Barz A, Culkin A, Kris MG, Artz D, et al. Long-term toxicity monitoring via electronic patient-reported outcomes in patients receiving chemotherapy. Journal of Clinical Oncology. 2007;25(34):5374-80.
